# P-142. Benefits and Pitfalls of Multiplex Gastrointestinal Polymerase Chain Reaction testing in a Veterans Affairs Hospital

**DOI:** 10.1093/ofid/ofaf695.369

**Published:** 2026-01-11

**Authors:** Jessica Schildkraut, Claire Lewis, Monique Thorne, Debra Noland, Akif Acay, George Psevdos

**Affiliations:** Northport VAMC, Northport, New York; Northport VAMC, Northport, New York; Northport VAMC, Northport, New York; Northport VAMC, Northport, New York; Northport VAMC, Northport, New York; Northport VA Medical Center, Northport, New York

## Abstract

**Background:**

In the US infectious gastroenteritis remains a significant cause for morbidity and a public health concern. While stool culture had been the standard tool for microbiological diagnosis, known for its time consuming and often lower yields, rapid diagnostic tests have now been developed. Multiplex gastrointestinal (GI) polymerase chain reaction (PCR) testing is commercially available as a rapid, sensitive method for detecting GI pathogens, bacteria, viruses, and parasites in a single stool sample. Nevertheless, challenges in interpreting results can appear especially in mixed infections where detections may not be clinically significant. While high sensitivity of testing is desirable, false positive results require careful interpretation. We reviewed the performance of GI PCR tests in our facility

**Table 1:**
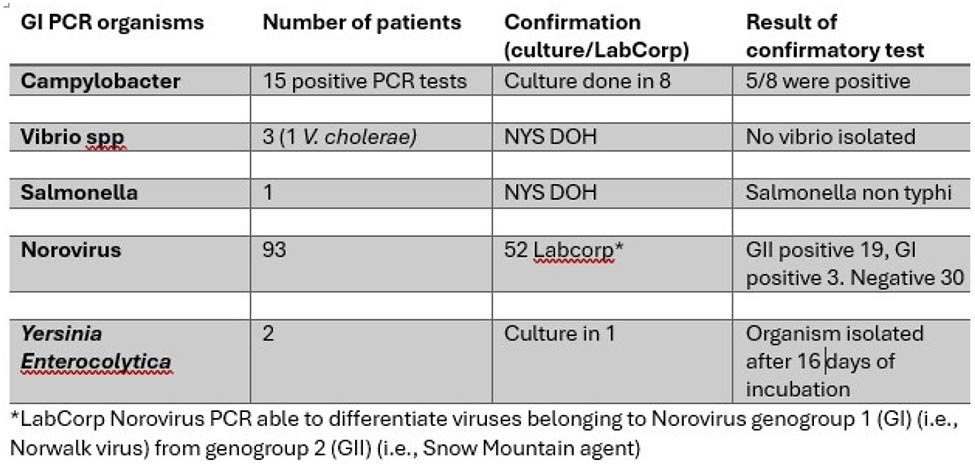
GI PCR results

**Table 2:**
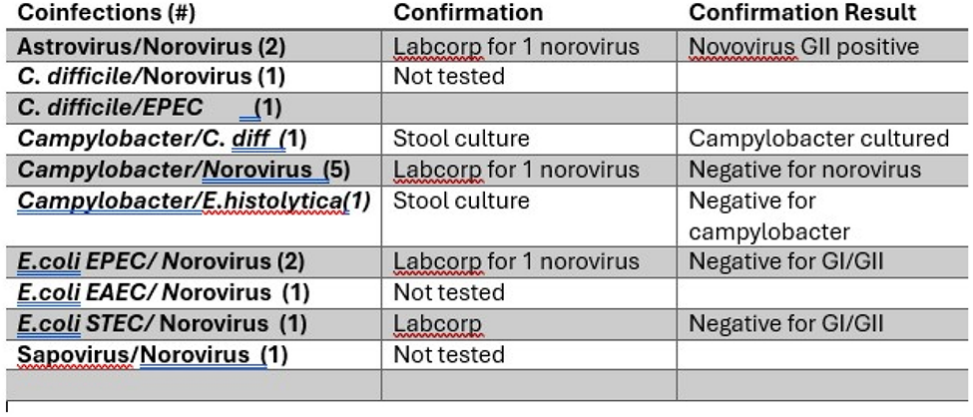
GI PCR COINFECTIONS

**Methods:**

Retrospective chart review from 2021 to 2024 of US Veterans at Northport Veteran Affairs Medical Center who had positive results on Biofire® FilmArray GI PCR. We focused on norovirus, *Campylobacter* and *Vibrio* results. Norovirus positive specimens were sent to LabCorp reference laboratory. *Campylobacter* results were compared to culture from Campylobacter selective agar plate. *Vibrio* and *Salmonella spp* results were sent for confirmation to our local department of health

**Results:**

111 GI PCR results were reviewed. The median age of the patients was 75 years (27-96). 8 were women. 64% Caucasian, 12% Black, 12% Hispanic. Table 1 lists the PCR results, Table 2 the coinfections. Of the 15 *Campylobacter* positive results, culture was done in 8 and cultured in 5. All 3 *Vibrio* results were not confirmed by culture. The 1 *Salmonella* result was confirmed as non-typhi. Of the 2 *Yersinia* results, one was cultured and was confirmed. Of the 93 Norovirus results, 52 were tested at LabCorp, 30 (58%) were negative. Genogroup II was most common (36%)

**Conclusion:**

A significant percentage of our Biofire GI PCR Norovirus positive results were false positive. Also, the *Vibrio* results were not reliable. On the other hand, it was more sensitive for *Campylobacter.* While the Biofire GI PCR is a valuable tool for detecting several gastrointestinal pathogens, it is important to be aware of the potential for false positive Norovirus and Vibrio results and to use clinical judgment and confirmatory testing when appropriate

**Disclosures:**

All Authors: No reported disclosures

